# AK2 Promotes the Migration and Invasion of Lung Adenocarcinoma by Activating TGF-β/Smad Pathway *In vitro* and *In vivo*

**DOI:** 10.3389/fphar.2021.714365

**Published:** 2021-09-22

**Authors:** Fangfang Cai, Huangru Xu, Daolong Zha, Xiaoyang Wang, Ping Li, Shihui Yu, Yingying Yao, Xiaoyao Chang, Jia Chen, Yanyan Lu, Zi-Chun Hua, Hongqin Zhuang

**Affiliations:** ^1^ The State Key Laboratory of Pharmaceutical Biotechnology, College of Life Sciences, Nanjing University, Nanjing, China; ^2^ School of Biopharmacy, China Pharmaceutical University, Nanjing, China; ^3^ Changzhou High-Tech Research Institute of Nanjing University and Jiangsu TargetPharma Laboratories Inc., Changzhou, China

**Keywords:** AK2, lung adenocarcinoma, EMT, tumor metastasis, TGF-β

## Abstract

Adenylate kinase 2 (AK2) is a wide-spread and highly conserved protein kinase whose main function is to catalyze the exchange of nucleotide phosphate groups. In this study, we showed that AK2 regulated tumor cell metastasis in lung adenocarcinoma. Positive expression of AK2 is related to lung adenocarcinoma progression and poor survival of patients. Knockdown or knockout of AK2 inhibited, while overexpression of AK2 promoted, human lung adenocarcinoma cell migration and invasion ability. Differential proteomics results showed that AK2 might be closely related to epithelial-mesenchymal transition (EMT). Further research indicated that AK2 regulated EMT occurrence through the Smad-dependent classical signaling pathways as measured by western blot and qPCR assays. Additionally, *in vivo* experiments showed that AK2-knockout in human lung tumor cells reduced their EMT-like features and formed fewer metastatic nodules both in liver and in lung tissues. In conclusion, we uncover a cancer metastasis-promoting role for AK2 and provide a rationale for targeting AK2 as a potential therapeutic approach for lung cancer.

## Introduction

Lung cancer is the leading cause of cancer-related mortality worldwide, with a poor 5-year survival rate of 15% (Siegel et al., 2018), among which non-small cell lung carcinoma (NSCLC) accounts for 75–80% of this disease (Meza et al., 2015). For early stage of lung cancer, when the tumor is confined to the lung or when there is slight local lymph node spread, surgery is an effective treatment. Unfortunately, about 70% of patients have locally advanced or extensive metastatic tumors when diagnosed, which is not suitable for surgical resection (Herbst et al., 2018). Although there are multiple treatment options including surgery, chemotherapy, radiation and targeted therapy, the prognosis of patients is still poor due to its high metastasis (Altorki et al., 2019). Therefore, discovering novel and functional targets is crucial.

Tumor metastasis is a complex process involving a series of cell activities, collectively referred to as the invasion-metastasis cascade process (Valastyan and Weinberg, 2011). Many studies have shown that epithelial-mesenchymal transition (EMT) plays an important role in tumor cell infiltration, migration and metastasis. It not only enhances cancer cell invasion and metastasis (Mani et al., 2008; Kalluri and Weinberg, 2009), but also enables them to acquire stem cell characteristics such as self-renewal, and promote cancer stem cell production. TGF-β, which is called transforming growth factor due to its role in cell transformation, not only participates in the EMT process of embryonic development (Mercado-Pimentel and Runyan, 2007), but also regulates tumor EMT process (Derynck and Zhang, 2003; Peinado et al., 2003; Micalizzi et al., 2009). Several evidences argue for the role of SMADs in TGF-β-induced EMT (Deckers et al., 2006; Vincent et al., 2009; Lamouille et al., 2014). After activation of the precursor, TGF-β forms a dimer, binds to its membrane surface receptor 2 (TβRⅡ), and then recruits two TGF-β receptors 1 (TβRⅠ). TβRⅠ is activated by TβRⅡ *via* phosphorylation and activates downstream signaling molecules Smad2 and Smad3, promoting the formation of a trimeric complex with Smad4. The complex enters the nucleus and interacts with DNA, up-regulating the expression levels of transcription factors Snail/Slug, Twist and ZEB1, down-regulating the expression levels of E-cadherin, thereby regulating EMT at the transcriptional level (Vincent et al., 2009; Lamouille et al., 2014).

Adenylate kinase (AK) is highly conserved across a wide range of organisms. It is a key enzyme for metabolic monitoring of cell adenine nucleotide homeostasis (Klepinin et al., 2020). It also directs AK→AMP→AMPK signal transduction, regulating cell cycle and cell proliferation, and transferring ATP energy from mitochondria to distribute energy among cellular processes. There are nine adenylate kinase isoenzymes (AK1-AK9) in different tissues and organs. Increasing evidences show that AK isoform network regulates various cellular processes, including cell motility and cell differentiation (Panayiotou et al., 2014). Adenylate kinase 2 (AK2), located in the space between membranes and slits, is essential for ATP export and mitochondrial nucleotide exchange (Dzeja and Terzic, 2009). AK2 deficiency can destroy cellular energy, leading to human diseases such as amyotrophic lateral sclerosis, severe combined immunodeficiency, deafness and cancer, and is embryonic lethal in mice (Fukada et al., 2004; Lagresle-Peyrou et al., 2009; Pannicke et al., 2009). Increasing evidences show that AK2 plays a vital role in tumor development. Hansel *et al.* found that AK2 is up-regulated in metastatic pancreatic endocrine tumors, which may be related to the promotion effect of AK2 on nucleotide signal transduction and metastasis (Hansel et al., 2004). Another study suggests that AK2 deficiency promotes the *in vitro* proliferation of breast cancer MCF-7 and C33A cells, and induces tumor formation in xenograft trials (Kim et al., 2014). However, although the relationship between AK2 and tumors has long been reported, the role and underlying mechanisms of AK2 in tumors, especially in non-small cell lung cancer, is still unclear.

In this study, we found that AK2 is closely related to the occurrence and development of non-small cell lung cancer, especially lung cancer cell metastasis. Moreover, AK2 induces EMT through the Snail/Smads/TGF-β signaling pathway in lung cancer cells, thereby affecting migration and invasion.

## Materials and Methods

### Plasmids and Reagents

Human AK2 encoding sequence was amplified from cDNA generated from A549 cell-derived mRNA as a template, and subcloned into a pcDNA3.1 vector (Invitrogen, Carlsbad, CA, United States). Cells were transfected with plasmid DNA using PolyJect™ reagent (Signagen, United States) as described previously (Yang et al., 2016), and harvested for the desired experiment until overexpression for 48 h.

### Cell Culture

NSCLC cell lines A549 and H1299, and HBE (Human bronchial epithelioid cells) were purchased from American Type Culture Collection (ATCC, Philadelphia, PA, United States). Cells were cultured in Dulbecco’s modified Eagle’s medium or RPMI-1640 (Thermo, United States) supplemented with 10% fetal bovine serum (FBS, Gibco, Australia) and 50 U/mL penicillin/streptomycin (Invitrogen, Carlsbad, CA, United States), in a humidified CO_2_ (5%) incubator at 37°C.

### siRNAs and Transfection

All synthetic siRNAs and negative controls (NCs) were purchased from Shanghai Gene Pharma Co. Ltd. For transfection, cells were transiently transfected with siRNAs or plasmids using lipofectamine 2000 (Invitrogen, United States) according to the manufacturer’s instructions. Immunoblotting analysis was used to further confirm cognate protein expression in all transfectants. The sequences of siRNAs for targets are listed as follows:

**Table:** 

siRNAs	Sequences
Negative Control-#1	5′-UUC​UCC​GAA​CGU​GUC​ACG​UTT-3′
Negative Control-#2	5′-ACG​UGA​CAC​GUU​CGG​AGA​ATT-3′
siAK2-#1	5′-GAA​GCU​UGA​UUC​UGU​GAU​UTT-3′
siAK2-#2	5′-CCA​GCC​AGU​UAG​UUA​UUC​ATT-3′
siAK2-#3	5′-GCA​CUU​GCU​UGA​UGU​AUC​UTT-3′

### Western Blotting Analysis

Whole-cell lysates of A549 and H1299 (approximately 5 × 10^6^ cells) were prepared with RIPA buffer (Santa Cruz Biotechnology) containing PMSF, orthovanadate, and protease inhibitors. Equal amounts of protein (40 µg) were separated by 12% SDS-PAGE gels and transferred onto nitrocellulose membranes blocked with 5% skim milk in TBS containing 0.1% Tween 20, and incubated with primary antibodies: anti-AK2, anti-GAPDH (Santa Cruz Biotechnology, Santa Cruz, CA), anti-pSmad2, anti-Smad2, anti-Smad3, anti-Smad4, anti-Fibronectin, anti-Vimentin, anti-Snail (Cell Signaling Technology, Beverly, MA, United States), anti-Actin (Abgent, San Diego, CA, United States), anti-SMA, anti-E-cadherin, anti-N-cadherin (BD Biosciences, CA, United States). Secondary antibodies were goat anti-rabbit or goat anti-mouse, coupled with horseradish peroxidase. The protein of interest was immunoplexed with the indicated primary antibody and corresponding secondary antibody. Bound antibodies were then visualized with ECL plus western blotting detection reagents (GE Healthcare). Signal intensity was quantified by densitometry using the software Image J (NIH, Bethesda, MD). All experiments were done in triplicate and performed at least three times independently.

### Quantitative Real-Time PCR

Total RNA was extracted from A549 and H1299 cells with TRIzol reagent (Invitrogen, United States) according to the manufacturer’s instructions. cDNA for quantitative real-time PCR (qRT-PCR) was generated using a ReverTra Ace® qPCR RT Kit (Toyobo). qRT-PCR was performed using a AceQ® qPCR SYBR® Green Master Mix (Vazyme, China) in a 96-well format following the manufacturer’s instructions. Primers were listed in Supplementary Table S1. Data were analyzed by StepOne 2.1 software (Applied Biosystems, United States) according to the manufacturer’s specifications. β-Actin was used as a control.

### LC-MS/MS Analysis and Bioinformatics Analysis

The LC-MS/MS analysis was performed as previously described (Yao et al., 2013). The protein concentration in the supernatant was determined using the bicinchoninic acid (BCA) method. Equal amount of protein (200 μg) was used for iTRAQ labeling according to the manufacturer’s instructions. Raw MS/MS data were analyzed by the Agilent G2721AA Spectrum Mill MS Proteomics Workbench (Rev A.03.03.078) in the UniProtKB/SWISS Prot database for protein identification. The network building tool MetaCore^TM^ version 5.4 (GeneGo) was used to establish potential signaling network.

### Tissue Microarray Analysis

Tissue microarrays (TMAs) of lung adenocarcinoma were purchased from Shanghai Outdo Biotech Co. Ltd. Samples include normal human lung tissue, lung adenocarcinoma paracancerous tissue, lung adenocarcinoma primary tumor, lung adenocarcinoma metastases, lung adenocarcinoma lymph nodes (negative), and lung adenocarcinoma lymph nodes (positive). Immunohistochemistry and H&E staining were used to analyze the diseased tissue microarray of clinical lung adenocarcinoma patients, and detect AK2 expression. The method for immunohistochemistry has been described previously (Jiang et al., 2019). Briefly, TMAs were incubated with an affinity-purified anti-AK2 antibody (Santa Cruz Biotechnology, Santa Cruz, CA) for 2 h. Diaminobenzidine tetrahydrochloride (DAB) was used as substrate, and the bound antibody was detected with polymerized HRP anti-rabbit IgG (Maixin, Fuzhou, China). Quantitative analysis of AK2 staining was applied with Image J software.

### Real-Time Cell Analysis of Cell Proliferation and Migration

Briefly, cells were digested and counted with Automated Cell Counter (Invitrogen, United States). 5 × 10^3^ cells of each group were seeded on modified 16-well plates (E-plate, Roche, Germany) and monitored with the xCELLigence RTCA DP instrument (Roche, Germany) for 24–48 h. Data collecting and analysis were in accordance with the manufacturer’s guidelines.

### Cell Proliferation Assay

Cell proliferation was measured by CCK8 assay. Cells transfected with Si-NC, Si-AK2, pcDNA3.1 and pcDNA3.1-AK2 were seeded on 96-well culture plates at a density of 2000 cells per well. The cells were incubated for 12, 24, 36 or 48 h, and cell viability was measured by a microplate spectrophotometer (Titertek Mul-tiskan MCC/340) equipped with a 450 nm filter. Each experiment was performed in quadruplicate and repeated at least three times.

### Cell Migration Assay

Cell migration assay was performed using transwell inserts (8.0 mm pore size, Millipore, Billerica, MA, United States). The cells were starved for 12 h prior to the experiment, then harvested and resuspended in a cell suspension diluted to 5×10^5^ cells/mL with serum-free DMEM containing 1% BSA. 200 μL of cell suspension was pipetted into the upper chamber, and the lower chamber was filled with 600 μL 10% FBS supplemented medium. After incubation at 37°C for 10 or 16 h, cells on the upper surface of the membrane were removed. The migrant cells attached to the lower surface were fixed in 4% paraformaldehyde at room temperature for 30 min, and stained for 30 min with a solution containing 1% crystal violet and 2% ethanol in 100 mM borate buffer (pH 9.0). The number of cells migrating to the lower surface was photographed in five fields under a microscope with a magnification of ×100. The chamber was then purged with 33% HAC (100 μL). After the crystal violet was completely dissolved and the cells were evenly distributed in the HAC solution, the assay was performed at 570 nm using a microplate reader (TECNA, Switzerland) and quantitative analysis was performed using GraphPad Prism V 8.0 software.

### Wound-Healing Assay

Cells were plated in 6-well culture plates to form cell monolayer (near 90% confluence). Wounds were made with a sterile P-200 micropipette to scrape off the cells. The wells were then washed three times with PBS to remove non-adherent cells for 36 h or 48 h. The progress of wound closure was monitored with microphotographs of ×10 magnification taken with light microscope (Carl Zeiss Axioplan 2) at the beginning and the end of the experiments after being washed with PBS.

### Immunofluorescence Staining

The immunofluorescence staining was performed as previously indicated (Cai et al., 2020). A549 cells were transfected with Si-NC and Si-AK2 interference fragments respectively. After 48 h, cells were harvested and immunostained with anti-E-cadherin (dilution 1:200) antibody. The cells were then incubated with Alexa Fluor 488-labeled secondary antibody (Thermo Fisher Scientific Inc.) for 1 h at room temperature. Images were acquired using an Olympus laser scanning confocal microscope (Olympus Optical Co., Tokyo, Honshu, Japan) and their optical densities were quantitatively calculated using Image J.

### Development of Tail Vein Injection Lung Cancer Model

A549 and A549 AK2-knockout (AK2-KO) cells (2 × 10^6^) suspended in 200 μL of PBS were injected into the tail vein of nude mice. Detailed treatment was described in Figure 5A. 30 days after tumor inoculation, all mice were euthanized to assess the number of spontaneous metastases in the lung and liver. The lungs and livers were harvested, fixed in 4% paraformaldehyde, and embedded in paraffin for pathological examination of H&E and immunofluorescence assay.

### H&E Assays

In parallel animal experiments (three groups in total, eight mice in each group), the establishment of tumors and drug treatment were the same as above. On the 30th day, the mice were euthanized. Lungs and livers of metastatic model mice were collected, fixed with 4% paraformaldehyde, and embedded in paraffin. Tissue sections (5 μm in thickness) were prepared according to standard protocols for hematoxylin/eosin (H&E) staining.

### Statistical Analysis

Data were presented as means ± SD. Comparisons within groups were done with a *t*-test with repeated measures; *p*-values indicated in figures are <0.05 (*), <0.01 (**), <0.005 (***), and <0.001 (****).

## Results

### AK2 was Up-Regulated in Human Non-Small Cell Lung Carcinoma and Associated With Poor Prognosis

**FIGURE 1:**
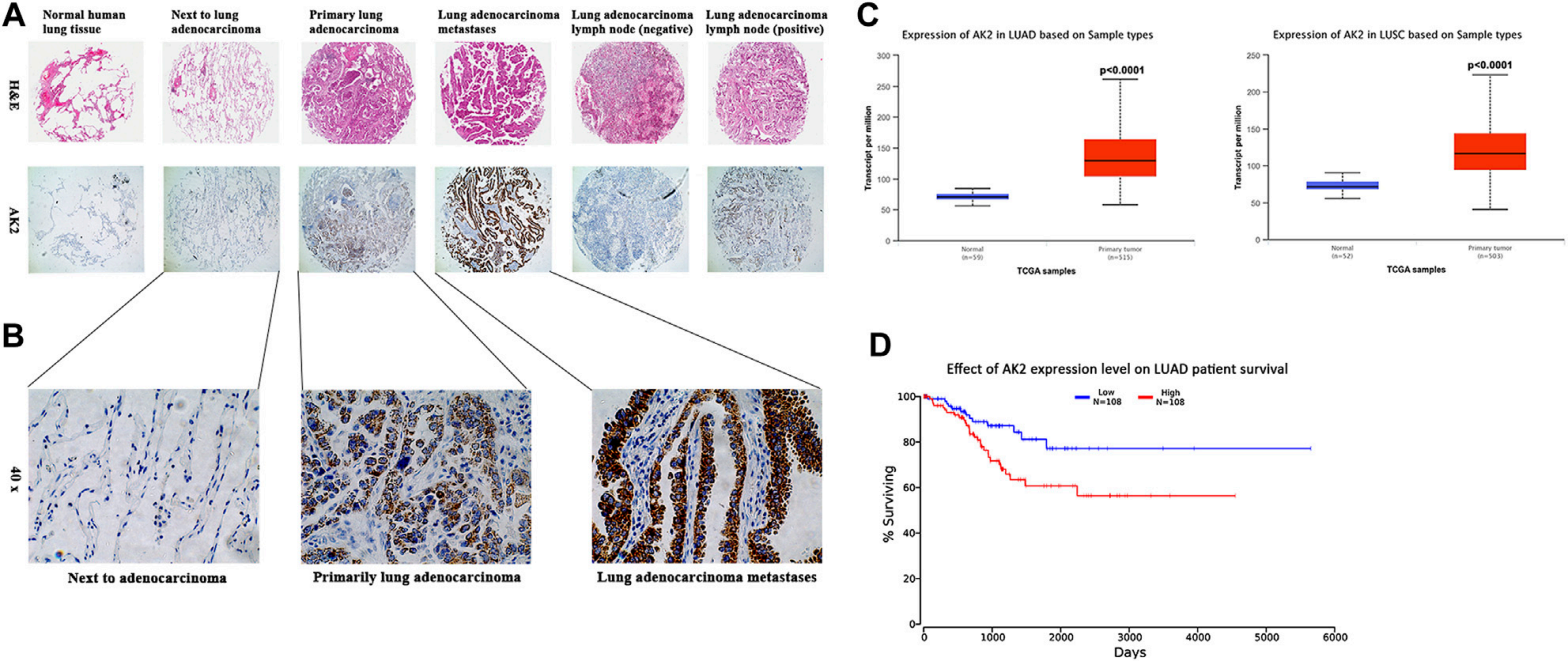
AK2 is up-regulated in human non-small cell lung carcinoma and is associated with poor prognosis. **(A)** AK2 expression was measured by H&E staining (100 ×) and immunohistochemical analysis (100 ×) on human tissue microarrays. Representative images were shown: normal lung tissue, lung adenocarcinoma adjacent tissue, lung adenocarcinoma primary tumor, lung adenocarcinoma metastasis, lung adenocarcinoma lymph node (negative), and lung adenocarcinoma lymph node (positive). **(B)** Further magnified AK2 expression in paracancerous, primary and metastatic carcinoma of the left frontal lobe (from lung adenocarcinoma) in patients with stage IV lung adenocarcinoma (200 ×). **(C)** Increased AK2 expression in Lung Adenocarcinoma (LUAD, left) and Lung Squamous cell Carcinoma (LUSC, right), analyzed in TCGA database separately. **(D)** Cancer survival analysis of AK2 expression in LUAD was assessed on Kaplan-Meier plotter. Meta-analysis based on biomarker assessment showed that high AK2 expression has a poor survival in human Lung adenocarcinoma. *p*-value is calculated using log-rank test.

To explore the role of AK2 in NSCLC progression, we first evaluated AK2 expression in pathological tissue microarray from lung adenocarcinoma patients. We found that AK2 was almost undetectable in normal lung tissues, while its expression level in lung cancer tissues gradually increased with the progresses of cancer (Figure 1A). Next, we examined AK2 level in the adjacent non-tumor tissues, primary lesion, and left frontal lobe metastasis from patients with stage IV lung adenocarcinoma by immunohistochemistry analysis. As shown in Figure 1B, the expression level of AK2 in tumor metastases tissues was markedly higher than that in adjacent non-tumor tissues and primary lesions (*p* < 0.001). To further confirm the expression levels of AK2 in non-small cell lung carcinoma, we downloaded and analyzed existing clinical data from The Cancer Genome Atlas (TCGA) database. Bioinformatic analysis showed that AK2 expression levels significantly increased in lung adenocarcinoma (LUAD) and lung squamous cell carcinoma (LUSC) than that in normal tissues (Figure 1C), and the *p*-values were all less than 0.0001. Then we divided the patients into a high AK2 expression group (AK2-high, moderate and strong; *n* = 108) and a low expression group (AK2-low, negative and weak; *n* = 108) to analyze the survival rate. Kaplan-Meier survival analysis showed that there was a statistically significant negative correlation between AK2 intensity and recurrence-free survival rate (Figure 1D). The above experimental results strongly indicated that AK2 was significantly up-regulated in human non-small cell lung cancer and closely related to the occurrence and development of tumors.

### Knockout of AK2 Significantly Inhibited Lung Cancer Cell Migration and Invasion

**FIGURE 2:**
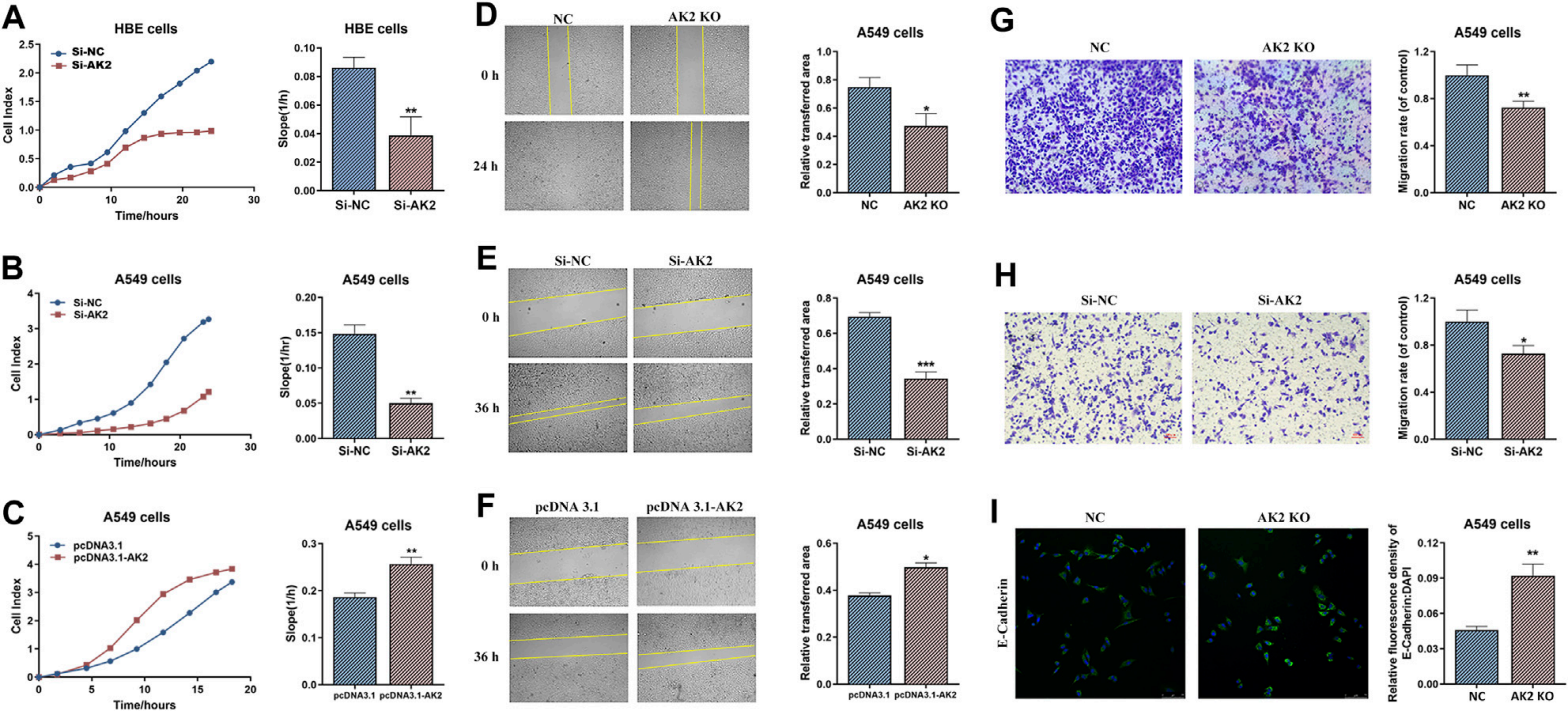
Knockout or knockdown of AK2 significantly inhibits lung cancer cell migration and invasion. **(A–C)** RTCA analysis on migration ability of AK2-knockdown HBE cells **(A)**, AK2-knockdown A549 cells **(B)** and AK2-overexpression (pRK5-HA-AK2) A549 cells **(C)**, respectively. Each bar is the mean of three independent experiments. Data are represented as mean ± S.D. **p < 0.01. **(D–F)** Wound healing assay on migration ability of AK2-knockout A549 cells **(D)**, AK2-knockdown (transfected with AK2 siRNA) A549 cells **(E)**, and AK2-overexpression (pRK5-HA-AK2) A549 cells **(F)**, respectively. Photographs were taken at 0, 24 or 36 h post scratch creation. Images shown are representative of three independent experiments. Data are represented as mean ± S.D. *p < 0.05, ***p < 0.005. **(G,H)** Transwell assay on invasion capability of AK2-knockout A549 cells **(G)** and AK2-knockdown (transfected with AK2 siRNA) A549 cells **(H)**. After 16 h, cells that migrated to the inferior membrane were stained and counted in five fields at a magnification of ×100. N = 3, bar = 50 μm. The experiments were carried out in triplicate and representative data were shown. Data are represented as mean ± S.D. *p < 0.05, **p < 0.01. **(I)** Immunofluorescence assay was used to detect the expression of EMT marker protein E-cadherin (200 ×) in AK2-knockout A549 cells.

The expression levels of AK2 in a series of lung cancer cells (H1299, A549, H446, and 95D cells) and HBE cell line (a normal control group) were analyzed by qPCR assay. The results showed that AK2 mRNA levels were substantially elevated in tumor cells and the contents were much higher in A549 and H1299 cells (Supplementary Figure S1). Therefore, A549 and H1299 cells were selected for further study. To explore the underlying mechanisms of AK2 in NSCLC, we constructed AK2-knockdown HBE cells, AK2-knockdown (AK2-KN) A549 cells and AK2-KO A549 cells (Supplementary Figure S2). The real-time cell analysis system (RTCA) was used to dynamically monitor the migration kinetic curve of A549 and HBE cells in the presence or absence of AK2. As shown in Figures 2A,B, the migration of HBE and A549 cells decreased significantly after AK2 knockdown compared with the control group (*p* < 0.01). Additionally, overexpression of AK2 induced a significant increase in the migration rate of A549 cells (Figure 2C; Supplementary Figure S2). Moreover, the dynamic proliferation kinetic curve of HBE and A549 cells were also analyzed by the RTCA system. The results showed that AK2 deficiency or knockdown significantly increased the proliferation rate of HBE and A549 cells (*p* < 0.01), while overexpression of AK2 in A549 cells led to a significant reduction in proliferation within 48 h (Supplementary Figure S3). Additionally, we further performed CCK8 assay to analyze the proliferation rate of AK2-KN or AK2-KO A549 cells. As shown in Supplementary Figure S4, knockdown of AK2 promoted the proliferation rate of A549 cells within 48 h; however, this effect would be reversed after the time point of 48 h. Therefore, we also employed the FACS analysis to detect the apoptosis of A549 and H1299 cells. In Supplementary Figure S5, the results showed that there was no significant difference between the control and AK2-KN A549 cells at the time point of 24 h. However, when it came to the time point of 72 h, knockdown of AK2 significantly promoted the apoptosis of A549 cells. The same phenomenon was also observed in AK2-KN H1299 cells. To sum up, the above results indicated that AK2 might contribute to cell movement and cell migration in lung cancer cells.

Next, we performed wound healing and transwell experiments to further analyze the role of AK2 in migration and invasion. According to wound-healing assay, A549 cells displayed low migrated capabilities in the absence of AK2 as indicated by the inability of AK2-KO and AK2-KN A549 cells to completely heal the wound scratch (Figures 2D,E). On the contrast, as shown in Figure 2F, over-expression of AK2 greatly increased the migration ability of A549 cells. Moreover, *in vitro* invasion assays also revealed similar results that down-regulation or deletion of AK2 markedly reduced the migration ability of A549 cells (Figures 2G,H). To further clarify whether cell migration regulated by AK2 is related to EMT, the protein level of E-cadherin (a typical epithelial cell marker) in AK2-KO A549 cells was analyzed through immunofluorescence staining. In response to the absence of AK2, E-cadherin protein level increased significantly, indicating that AK2 might regulate EMT in lung cancer cells (Figure 2I).

### AK2 Regulated Lung Cancer Cell Migration Through EMT Process

**FIGURE 3:**
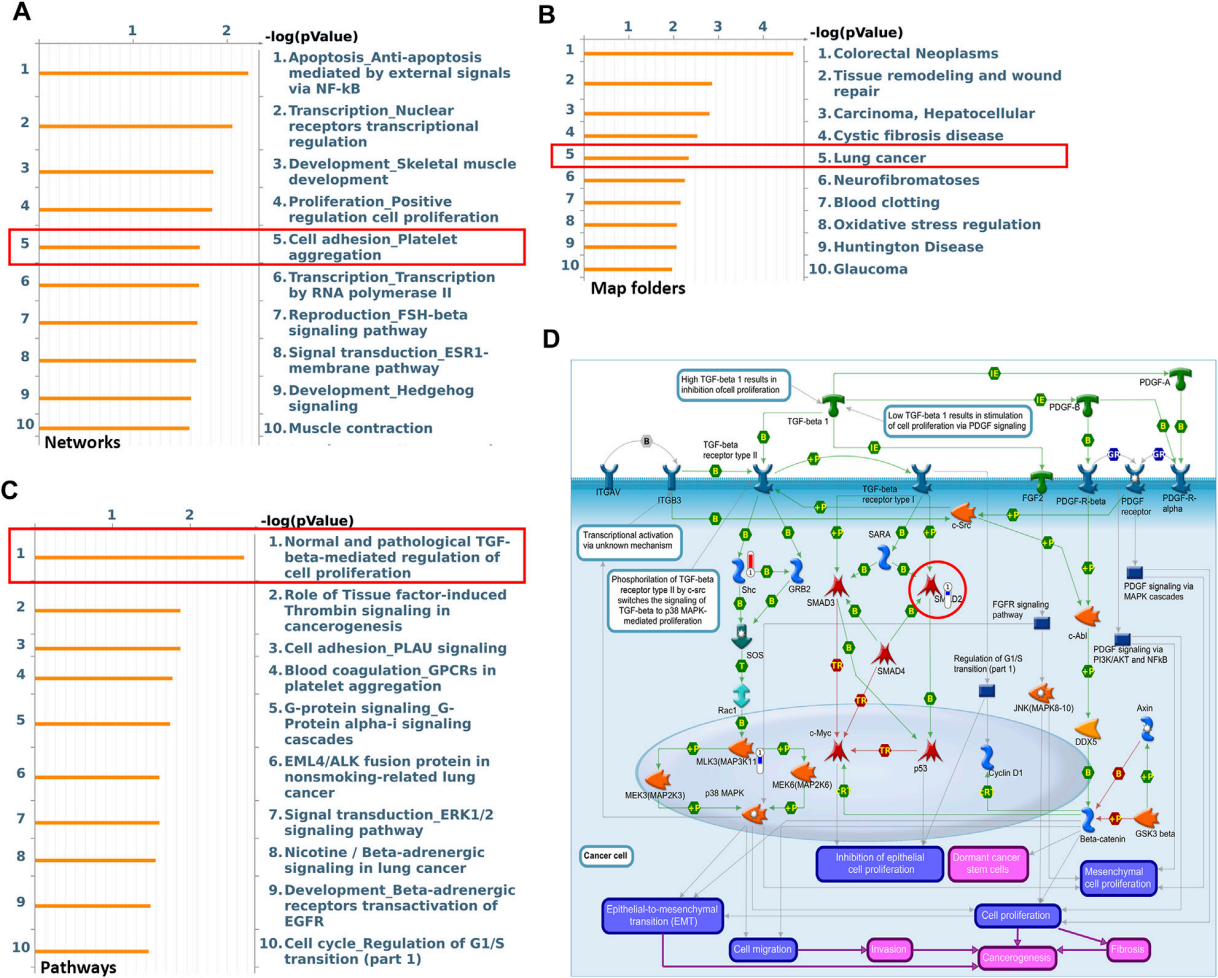
AK2 participates in lung cancer cell migration by regulating the EMT process. **(A,B)** Top ten most significant process **(A)** and Map Folders **(B)** predicted by Metacore^TM^ genego analysis. The results were ordered by -log10 of the *p* value of the hypergeometric distribution. **(C)** The top ten most significant pathway maps related to the lung-cancer map folders. **(D)** Pathway map related to TGF-β/EMT/Smads signaling axis within the top ten most significant pathways. Thermometers with blue or red next to symbols show proteins identified in LC-MS/MS detection: red color represents proteins up-regulated in AK2-knockout A549 cells compared to control group; blue color represents the down-regulated proteins.

High throughput proteomic approach was further performed to analyze the protein expression levels between AK2-deficient and control A549 cell lines. Differential proteomics analysis found that compared with the control A549 cells, there were 453 differentially expressed proteins (changes more than 1.4-fold) in response to AK2-knockout. The MetaCore^TM^ software was then used to analyze the differential proteomics and the underlying signal pathways. MetaCore^TM^ process network analysis clustered the differential networks from DEPs results, among which cell adhesion and migration showed a high score (Figure 3A). Next we also clustered the potential pathway maps in response to the absence of AK2 though the MetaCore^TM^ -Map folders software. As shown in Figure 3B, a series of signal pathways potentially regulated by AK2 was clustered and the lung cancer-related signaling pathways showed a high score, among which we found that AK2 might be involved in the TGF-β signaling pathway in lung cancer (Figure 3C). Transforming growth factor β (TGF-β) is a secreted cytokine that regulates proliferation, migration and differentiation of many cell types. TGF-β functions as a tumor promotor by stimulating tumor cells to undergo EMT process, leading to metastasis and chemotherapy resistance (Hao et al., 2019). Potential regulatory mechanisms of AK2 on stimulation of TGF-β signaling in lung cancer was also clustered by the MetaCore^TM^ pathway mapping tool, which is shown in Figure 3D. Several proteins involved in this pathway were found to be differentially expressed, such as Smad2, Smad3 and so on. Moreover, we found that the EMT process also participated in the regulation of lung cancer migration in AK2-KO cells (Figure 3D). Considering the important role of EMT in tumor migration and invasion, we speculated that AK2 might affect lung cancer cell migration ability through the TGF-β/EMT process.

### AK2 Promoted EMT Process *via* Snail/Smads/TGF-β Signaling

**FIGURE 4:**
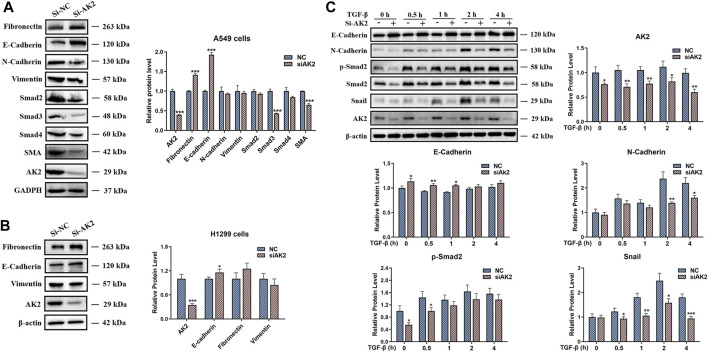
AK2 induces Snail/Smads/TGF-β signaling to activate EMT process. **(A,B)** A549 **(A)** or H1299 cells **(B)** were transfected with AK2 siRNA/NC for 48 h. The cell lysates were performed by western blotting analysis with indicated antibodies. **(C)** A549 cells were transfected with AK2 siRNA/NC for 24 h respectively, then stimulated with TGF-β for 0, 0.5, 1, 2, 4 h, respectively. Western blotting analysis was performed to detect the levels of E-cadherin, N-cadherin, *p*-Smad2, Smad2, Snail and AK2 respectively. β-actin was used as loading control. Band intensity was quantified by densitometry using the software Image J. Data are represented as mean ± S.D. *p < 0.05; **p < 0.01; ***p < 0.005. Each bar is the mean of three independent experiments.

EMT is regarded as a significant feature of most cancers and plays an important role in cancer migration and invasion (Heerboth et al., 2015; Aiello and Kang, 2019). Based on the role of AK2 in the EMT process and in the progression of NSCLC, we next examined the expression levels of EMT markers in lung cancer cells. Western blotting and qPCR assays were used to detect the protein and mRNA levels of EMT markers regulated by AK2. As shown in Figure 4A; Supplementary Figure S6, the protein and mRNA levels of EMT-typical markers including E-cadherin and Fibronectin were significantly up-regulated, while N-cadherin and Vimentin were down-regulated in AK2-KN A549 cells. Additionally, the expression levels of Smad2/Smad3/Smad4 were significantly decreased in A549 cells in response to AK2 knockdown (Figure 4A; Supplementary Figure S6). Moreover, knockdown of AK2 significantly down-regulated the expression levels of pSmad2 and pSmad3 (Supplementary Figure S7). Furthermore, the regulation of AK2 on the expression levels of Fibronectin, Vimentin and E-cadherin in H1299 cells was similar to that in A549 cells (Figure 4B; Supplementary Figure S8). According to the above differential proteomics analysis, AK2 might be involved in regulating the EMT pathway induced by TGF-β, which is the main inducer of EMT process through the Smad-dependent or Smad-independent pathways (Seoane and Gomis, 2017; Hao et al., 2019). Thus, TGF-β was used to induce EMT process and the effects of AK2 on the EMT process under dynamic conditions were further analyzed. A549 cells were transfected with Si-NC or Si-AK2 interference fragments for 24 h and then treated with TGF-β for 0, 0.5, 1, 2 and 4 h, respectively. Western blotting analysis was used to detect the phosphorylation of Smad2 and the expression levels of other EMT markers. As expected, the result of western blot showed a drastically increased phosphorylation level of Smad2 in A549 cells after treatment with TGF-β in a time-dependent manner (Figure 4C). However, the phosphorylation of Smad2 and the EMT process induced by TGF-β were significantly inhibited when AK2 was suppressed in A549 cells (Figure 4C). Moreover, the similar results were also observed in H1299 cells, in which down-regulation of AK2 markedly decreased the EMT process markers E-cadherin and Vimentin (Supplementary Figure S9). These results suggested that AK2 could promote the lung cancer cell migration through Smad2-mediated TGF-β-induced EMT process.

Otherwise, several researches have reported that FADD, the key adaptor protein in the apoptosis signaling pathway, was correlated with both tumor aggressiveness and tumor progression (Cimino et al., 2012; Fan et al., 2013; Pattje et al., 2013). Moreover, AK2 is one of the negative regulators of FADD phosphorylation (Kim et al., 2014). As shown in Supplementary Figure S10, the protein expression level of phosphorylated FADD increased significantly in AK2-knockout A549 cells. Additionally, we have recently provided the evidence that FADD regulated the migration ability of mouse embryonic fibroblasts (MEF) cells by affecting the phosphorylation and stability of PKCα (Cheng et al., 2012). PKCα mediates the down-regulation of its substrate ZEB1 to suppress the mesenchymal phenotype, including inhibition of cell migration and invasiveness (Llorens et al., 2019). Similar to previous research results in MEF cells, we found that the protein expression levels of phosphorylated PKCα were significantly down-regulated in AK2-KO A549 cells. Moreover, we further detected the protein and mRNA expression levels of PKCα in AK2-KN A549 cells. As shown in Supplementary Figure S10B,C, after transfection with AK2 siRNA for 48 h, the protein expression level of phosphorylated PKCα was significantly downregulated. However, there was a slight down-regulation on the total protein expression level of PKCα after AK2 knockdown. Additionally, we found that the mRNA level of PKCα was significantly down-regulated after transfection with AK2 siRNA in A549 cells (Supplementary Figure S10C). Based on the above results, we concluded that AK2-mediated activation of FADD/PKCα pathway may also contribute to the activation of EMT progress.

### AK2 Promoted Lung Cancer Cell Invasion *In vivo*

**FIGURE 5:**
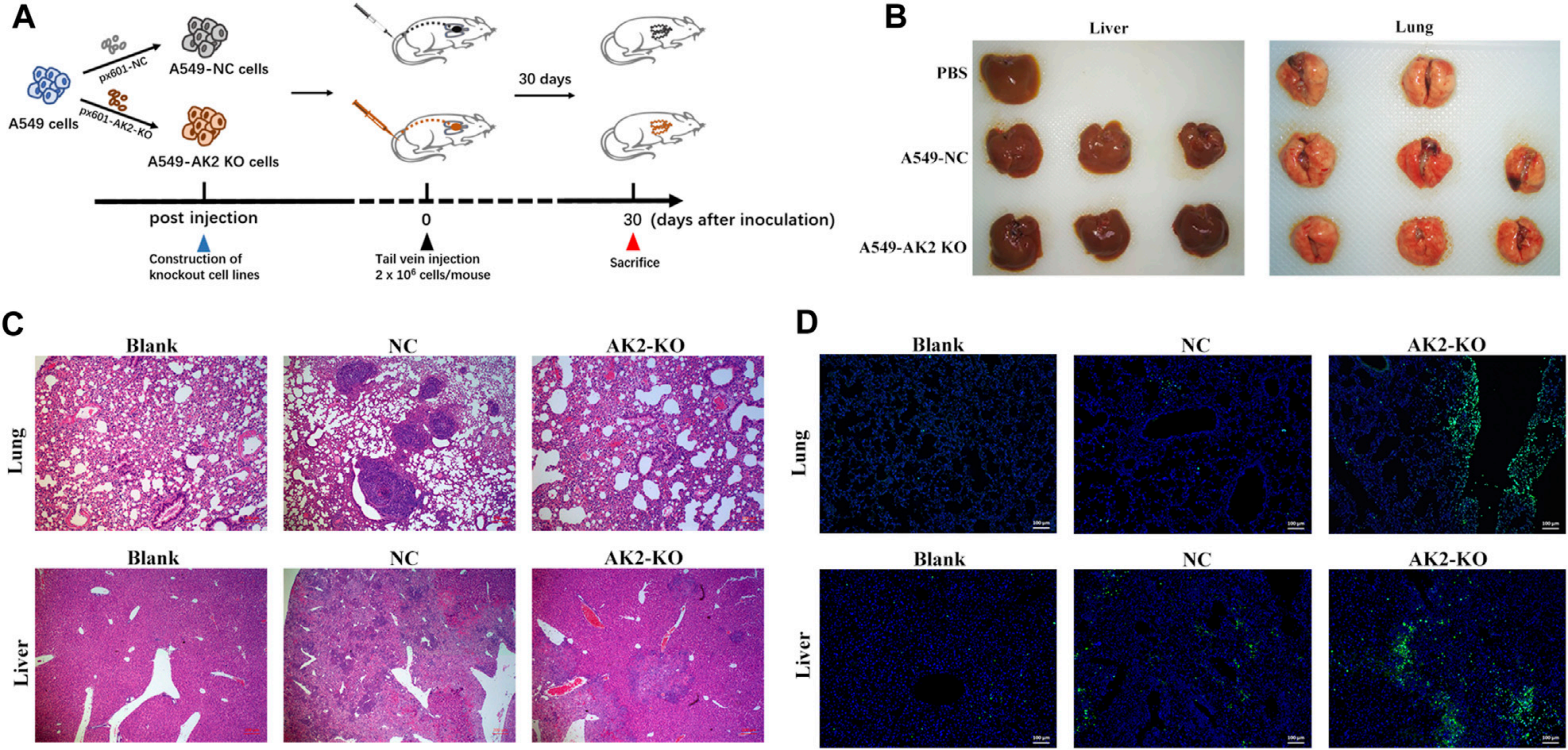
AK2 promotes lung cancer cell invasion *in vivo*. **(A)** The schematic diagram of establishing a lung cancer metastasis model by tail vein injection. **(B)** Representative whole organ imaging of liver **(left)** and lung **(right)** in nude mice injected with A549-NC or A549-AK2 KO cells. **(C)** Representative paraffin sections of lung and liver tissues stained with H&E in nude mice injected with A549-NC or A549-AK2 KO cells. Bars indicate 100 μm. **(D)** Representative immunofluorescence images of labeled epithelial marker (E-cadherin) of lung and liver in nude mice injected with A549-NC or A549-AK2 KO cells. Bars indicate 100 μm *n* = 6 mice in each group.

Given that knockout of AK2 significantly inhibited lung cancer cell migration and invasion, we next investigated the effects of AK2 knockout *in vivo* using the mice passive lung metastasis model (Figure 5A). A549-NC and A549-AK2 KO cells were injected into nude mice through tail vein, and then the mice were sacrificed 30 days later to obtain lung and liver tissues (Figure 5B). Following orthotropic transplantation of A549-NC and A549-AK2 KO cells into nude mice, all groups successfully formed tumors. Pulmonary metastases occurred in both the A549-NC and A549-AK2 KO groups. In addition, the representative lung and liver metastasis are shown in Figure 5C. H&E staining of lung tissue sections showed that there were obvious tumor metastases in the lungs of mice injected with A549-NC cells, accompanied by a large number and areas of nodules. Interestingly, this phenomenon was significantly improved after AK2 knockout; the number of lung nodules was significantly reduced and almost no significant lung nodules were observed. The tumor metastasis in the liver was also consistent with the results obtained in the lung. AK2 deficiency markedly reduced liver nodules as compared to the control group (Figure 5C). Immunofluorescence assay was further performed, showing that A549-AK2 KO cells exhibited overexpressed epithelial markers, E-cadherin, as compared with the control (Figure 5D). Collectively, our data showed that AK2 is highly expressed in lung cancer and promotes lung cancer cell migration and invasion, which may be caused by the involvement of AK2 in the EMT process regulated by TGF-β/Smad signaling pathway.

## Discussion

Tumor cell migration is the main cause of tumor proliferation, recurrence, drug resistance and even death. Inhibiting tumor cell metastasis and proliferation is an important direction to treat and limit tumor development, especially in lung cancer. AK2 senses AMP, activates metabolic signaling processes and maintains energy homeostasis in the cell. Although earlier work has demonstrated the biochemical differences of AK2 in lung cancer cases, the molecular events that regulate tumor growth and metastasis are still unknown.

In this study, we firstly conducted immunohistochemical experiments on lung adenocarcinoma tumor tissues of clinical patients, and found that AK2 intensity in the left frontal lobe metastatic carcinoma (from lung adenocarcinoma) tissue were significantly increased. Furthermore, there was a statistically significant inverse association between AK2 expression levels and recurrence-free survival, and high expression of AK2 significantly promoted the progress of lung cancer. Next *in vitro* experiments found that knockout or knockdown of AK2 could reduce lung cancer cells migration speed. However, it is rarely reported that AK2 affects tumor cell migration, and the specific mechanism is still unclear. Therefore, we used a high-throughput differential proteomics technology to analysis proteins changed between A549-AK2 KO and A549-NC cells. Eventually, 453 differentially expressed proteins were detected. To characterize the molecular pathways affected by AK2, Metacore^TM^ pathway mapping tool was used to show that AK2 might regulate the metastasis of tumor cells through EMT process. EMT is a key cellular phenomenon that regulates tumor metastasis. It is characterized by tumor cells losing typical epithelial characteristics, including cell polarity and intercellular adhesion, and gaining mesenchymal properties (Aiello and Kang, 2019). Interestingly, EMT implicates key cancer-related death processes, including cell migration and invasion. Currently, many studies have confirmed that EMT plays a decisive role in tumor development and metastasis (Aiello and Kang, 2019; Cho et al., 2019; He et al., 2019; Tulchinsky et al., 2019).

As the most important marker of EMT phenomenon, E-cadherin is very important for maintaining the stability of cell connection and adhesion (Chao et al., 2010). The results of immunofluorescence experiments showed that after deletion of AK2, E-cadherin level was significantly up-regulated, indicating that AK2 did participate in EMT regulation. E-cadherin up-regulation in response to AK2 deficiency inhibited EMT occurrence. Subsequent western blotting and qPCR experiments on A549 and H1299 cells showed that the mRNA and protein levels of E-cadherin increased significantly, while the expression levels of Vimentin and N-Cadherin decreased when the expression level of AK2 was suppressed. Therefore, we conclude that AK2 could promote EMT occurrence, thereby accelerating cell migration.

Recently, many investigations have revealed that inhibiting EMT induced by TGF-β in A549 cells and HK-2 cells significantly improves tumor migration (Liao et al., 2015; Bai et al., 2019). Proteomics results in our study show that AK2 may be involved in EMT process induced by TGF-β. The signaling pathway where TGF-β regulates EMT occurrence is divided into Smad-dependent classic signaling pathway and non-Smad-dependent signaling pathway (Derynck and Zhang, 2003). Smad3 is a key protein in the former. It can be activated together with Smad2 and combine with Smad4 to form a trimeric complex. The complex undergoes nuclear translocation and interacts with DNA to up-regulate the expression levels of transcription factors Snail/Slug, Twist, and ZEB1, down-regulate the expression levels of E-cadherin, and then regulate EMT at transcriptional level. In our study, we found that Smad3 expression was significantly down-regulated after AK2 silence, which further verified that AK2 regulates EMT occurrence through the Smad-dependent classical signaling pathway. Then we used TGF-β to stimulate cells to induce EMT, and detected changes in the expression levels of EMT-related markers. Compared with control group, Smad2 phosphorylation was inhibited to a certain extent after AK2 was silenced. Smad2 phosphorylation is an important process of the Smad signaling pathway. It might be preliminarily inferred that AK2-knockdown might improve TGF-β-induced EMT process of tumor cells through the Smad signaling pathway. FADD is a key regulator in cell biology process, including apoptosis, autophagy, cell necrosis, cell migration and so on (Mouasni and Tourneur, 2018). It was reported that AK2 activates DUSP26 phosphatase activity to dephosphorylate FADD and regulate the phosphorylation level of FADD (Kim et al., 2014). Moreover, FADD regulates the phosphorylation and stability of PKCα to participate in cell migration ability (Cheng et al., 2012; Llorens et al., 2019). In this study, we found that knockout of AK2 increased the phosphorylation levels of FADD and decreased the phosphorylation levels of PKCα in A549 cells, thereby participating in the EMT process.

AK2 is an isoenzyme of the AK family and may have momentous extra-mitochondrial functions, including cell proliferation and cell apoptosis (Klepinin et al., 2020). Recent studies have shown that AK2 can be transferred to the cytoplasm to participate in the mitochondrial apoptosis pathway (Lee et al., 2007; Maslah et al., 2021). In our study, we found that knockdown of AK2 significantly increased the cancer cell apoptosis and suppressed the proliferation in A549 and H1299 cells. The same phenomenon was also reported by Liu et al. (2019). In their research, they found that knockdown of AK2 could markedly suppress the proliferation and facilitate the apoptosis in lung adenocarcinoma cells (Liu et al., 2019). In addition, the inhibitory effects of AK2 on cell apoptosis have also been reported in hematopoietic stem and progenitor cell (HSPC) (Rissone et al., 2015), T-ALL cells (Maslah et al., 2021) and others. It is worth noting that AK2 might play different roles in apoptotic process in different cell types. For instance, another research group reported that knockdown of AK2 attenuated apoptosis by forming a complex with FADD and caspase 10 in HeLa cells (Lee et al., 2007). Also, in the study by Maslah et al. (2021), the authors found that AK2 depletion significantly promoted T-ALL cells apoptosis rather than BCP-ALL cells. Therefore, the relationship between AK2 and cell apoptosis is still unclear, and needs to be further explored.

In our study, the majority of the results reinforce the notion that AK2 is a positive regulator of NSCLC progression. AK2 promoted human lung cancer cells invasion *in vitro* and *in vivo*, and overexpression of AK2 fostered this progression by inducing EMT. To gain insight into the role of EMT played in lung cancer metastasis induced by knocking out of AK2, we evaluated the metastatic potential by lung colony formation efficiency using nude mice model. Consistent with the results obtained from *in vitro* experiments, knocking out of AK2 significantly inhibited lung metastasis and up-regulated the expression levels of E-cadherin *in vivo*. Therefore, it can be inferred that the knockout of AK2 in A549 cells significantly inhibits the EMT process, thereby inhibiting the metastatic activity of A549 cells and reducing the tumor areas that gather in the liver and lungs of mice.

**FIGURE 6:**
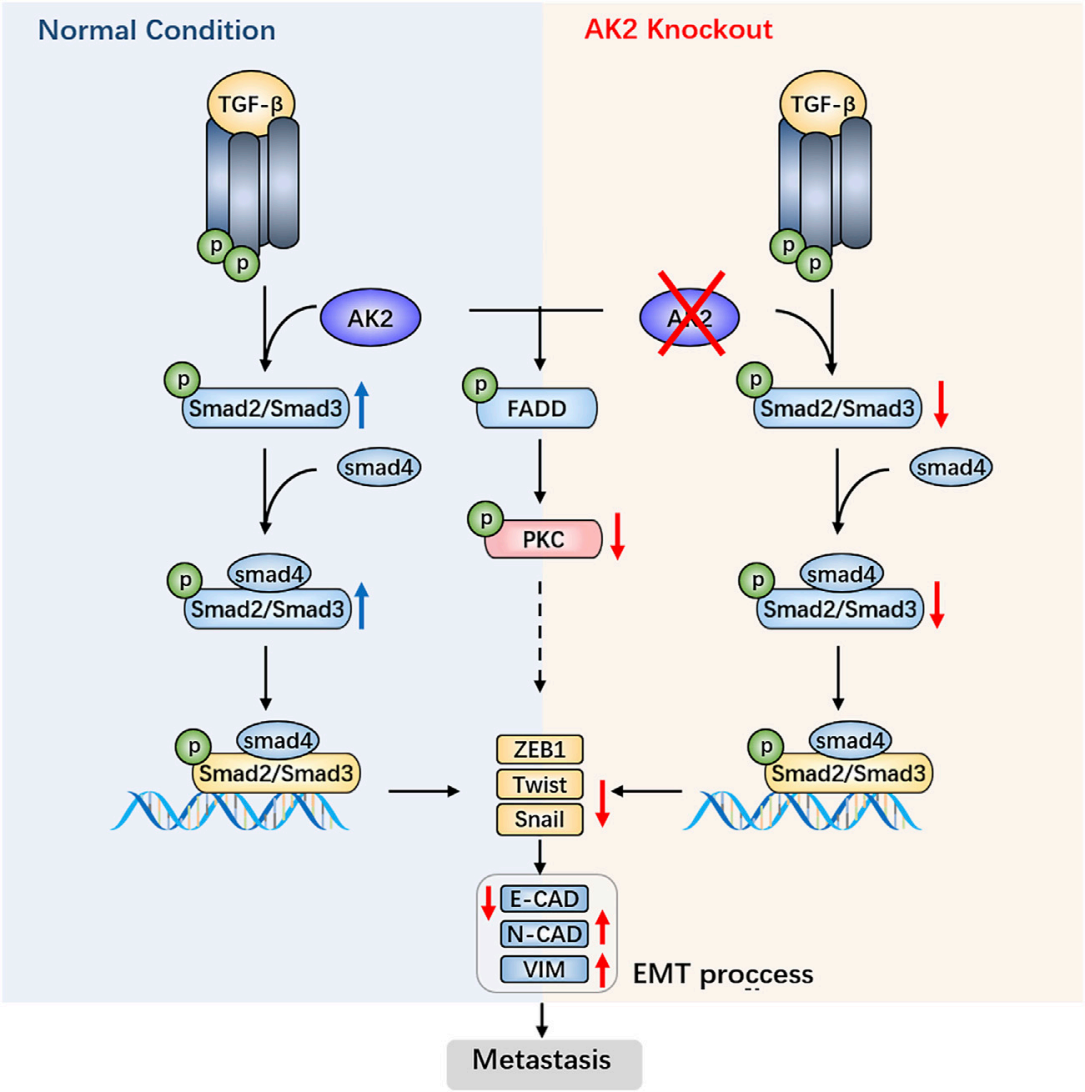
AK2 promotes the migration and invasion of lung adenocarcinoma by activating TGF-β/Smad pathway.

In conclusion, we provide insight into the biological function of AK2 in human lung adenocarcinoma and demonstrate that AK2 overexpression activates the TGF-β/Smad3/Smad2/Smad4 signaling pathway, leading to enhanced invasion *via* EMT (Figure 6). Consequently, targeting AK2 in a subset of lung cancers would be an optimal therapeutic strategy, and AK2 may be a biomarker for predicting responsiveness to lung cancer metastasis treatment.

## Data Availability

The original contributions presented in the study are included in the article/Supplementary Material, further inquiries can be directed to the corresponding authors. Besides, the mass spectrometry proteomics data presented in the study are deposited in the ProteomeXchange repository with the dataset identifier PXD028247.
