# Supplementary material for: AK2 Promotes the Migration and Invasion of Lung Adenocarcinoma by Activating TGF-β/Smad Pathway In vitro and In vivo
Source: Front Pharmacol. 2021 Sep 22;12:714365. doi: 10.3389/fphar.2021.714365 (PMC8493805; doi:10.3389/fphar.2021.714365)
Supplement: Supplementary file 1 [file DataSheet1.docx]

**AK2 promotes the migration and invasion of lung adenocarcinoma by activating TGF-β/Smad pathway *in vitro* and *in vivo***

Fangfang Cai^1,#^, Huangru Xu^1,#^, Daolong Zha^1^, Xiaoyang Wang^1^, Ping Li^1^, Shihui Yu^1^, Yingying Yao^1^, Xiaoyao Chang^1^, Jia Chen^1^, Yanyan Lu^1^, Zi-Chun Hua^1,2,3,*^, Hongqin Zhuang^1,*^

**Table S1 Primers for quantitative real-time PCR.**

| **Primers** | **Sequences** |
| --- | --- |
| h-AK2-F | 5’-GCAGAACCCGAGTATCCTAAAGG-3’ |
| h-AK2-R | 5’-TTCCCAGCATCCATAGTTGCC-3’ |
| h-Ecadherin-F | 5’-GACAACAAGCCCGAATTC-3’ |
| h-Ecadherin-R | 5’-GGAAACTCTCTCGGTCCA-3’ |
| h-Vimentin-F | 5’-GAGAACTTTGCCGTTGAAGC-3’ |
| h-Vimentin-R | 5’-GCTTCCTGTAGGTGGCAATC-3’ |
| h-Snail-F | 5’-TTCTCACTGCCATGGAATTCC-3’ |
| h-Snail-R | 5’-GCAGAGGACACAGAACCAGAA-3’ |
| h-PKCα-F | 5’-GTCCACAAGAGGTGCCATGAA-3’ |
| h-PKCα-R | 5’-AAGGTGGGGCTTCCGTAAGT-3’ |
| h-Fibronectin-F | 5’-ACTGGATGCTCCCACTAAC-3’ |
| h-Fibronectin-R | 5’-GTCGTCCTCTTCGGGTAA-3’ |
| h-β-actin-F | 5’-CATCGACACGGCATCGTCA-3’ |
| h-β-actin-R | 5’-TAGCACAGCCTGGATAGCAAC-3’ |

**Supplementary Figures**

**Figure S1 AK2 is substantially elevated in A549 and H1299 cells.**


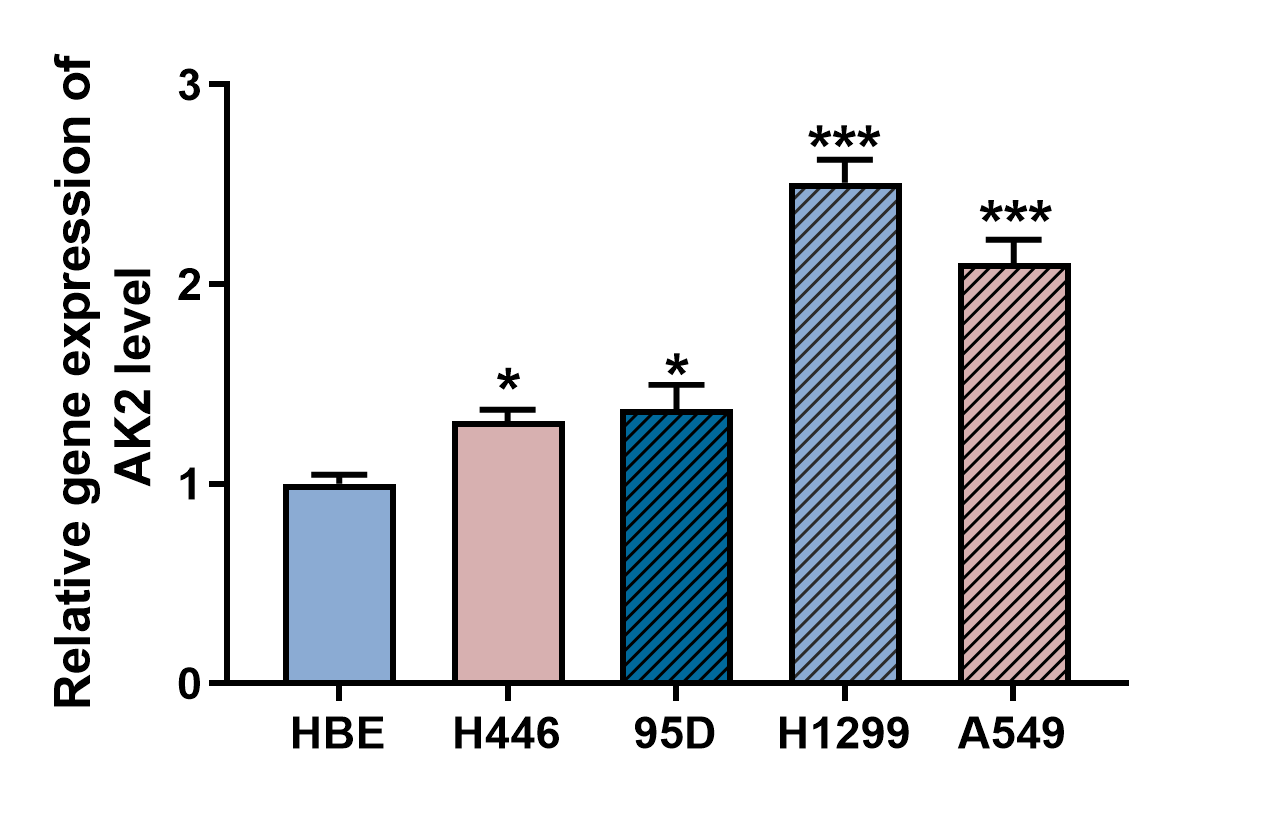


**Figure S1** **AK2 is substantially elevated in A549 and H1299 cells.** qPCR analysis was used to detect AK2 mRNA levels in HBE, H446, 95D, H1299 and A549 cells. **p* < 0.05, ****p* < 0.005*.* Data are expressed as mean ± S.D. of three independent experiments.

**Figure S2 Construction and validation of AK2-knockout, AK2-knockdown and AK2-overexpression cell lines.**


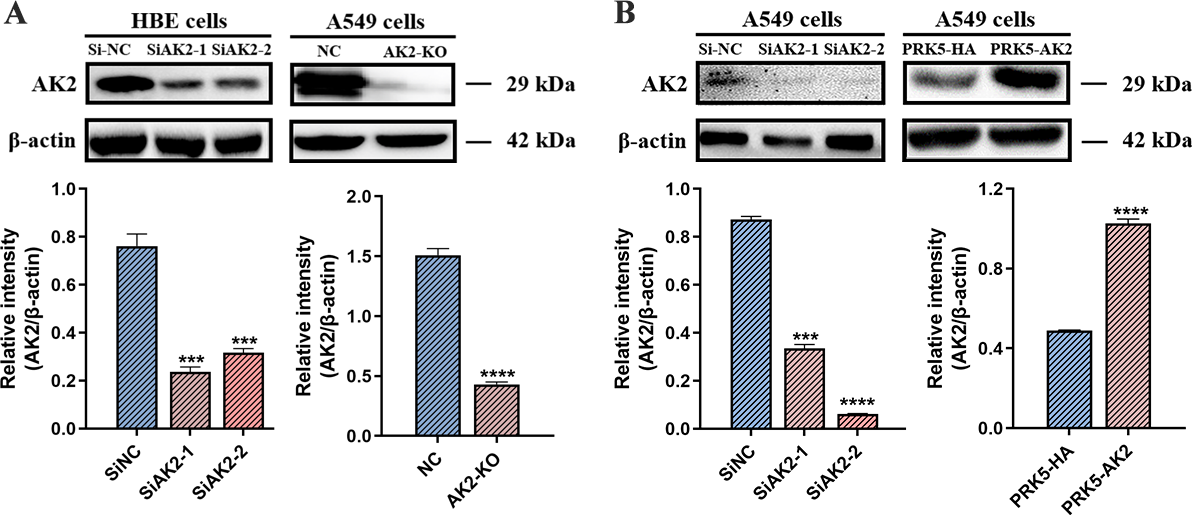


**Figure S2 Construction and validation of AK2-knockout, AK2-knockdown and AK2-overexpression cell lines. (A)** Detection of AK2 expression levels by Western Blot analysis in HBE control and AK2-knockdown HBE cell lines, A549 NC and A549 AK2-KO (AK2-knockout) cells. **(B)** Detection of AK2 expression levels by Western Blot analysis in A549 cells transfected with si-NC or si-AK2 interference fragment, PRK5-HA or PRK5-AK2 vectors. Data are represented as mean ± S.D. ****p* < 0.005, *****p <* 0.001*.*

**Figure S3 Effects of AK2 on lung cancer cell proliferation within 48 h.**


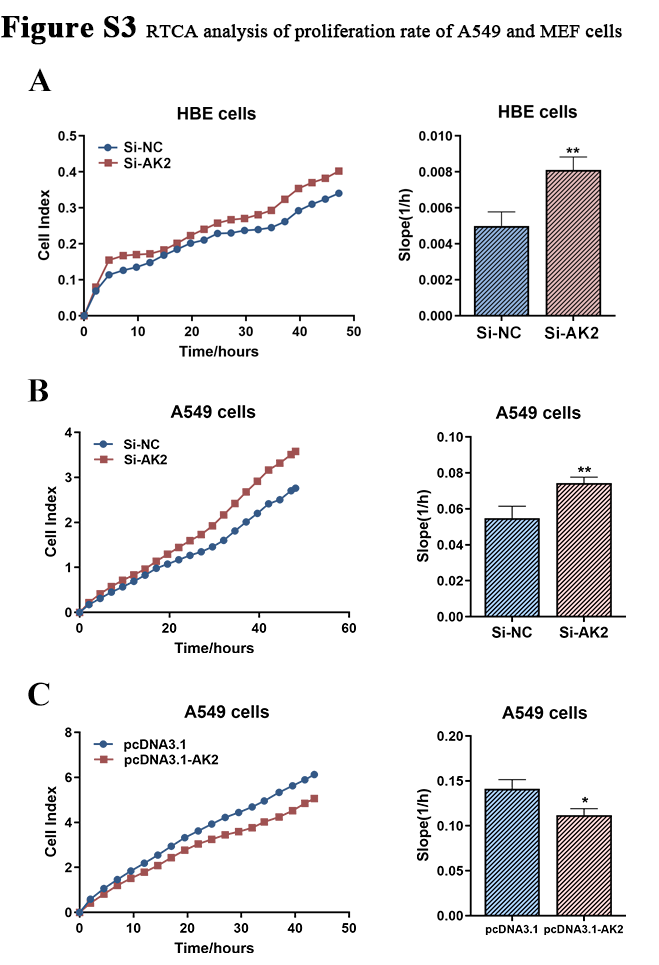


**Figure S3 Effects of AK2 on lung cancer cell proliferation within 48 h.** RTCA analysis of proliferation of HBE control and AK2-knockdown HBE cell lines (**A**), control and AK2-knockdown A549 cell lines (**B**), or control and AK2-overexpression A549 cell lines (**C**) within 48 h. Data are represented as mean ± S.D. **p* < 0.05, ***p* < 0.01.

**Figure S4 Effects AK2 knockout or knockdown on lung cancer cell proliferation.**


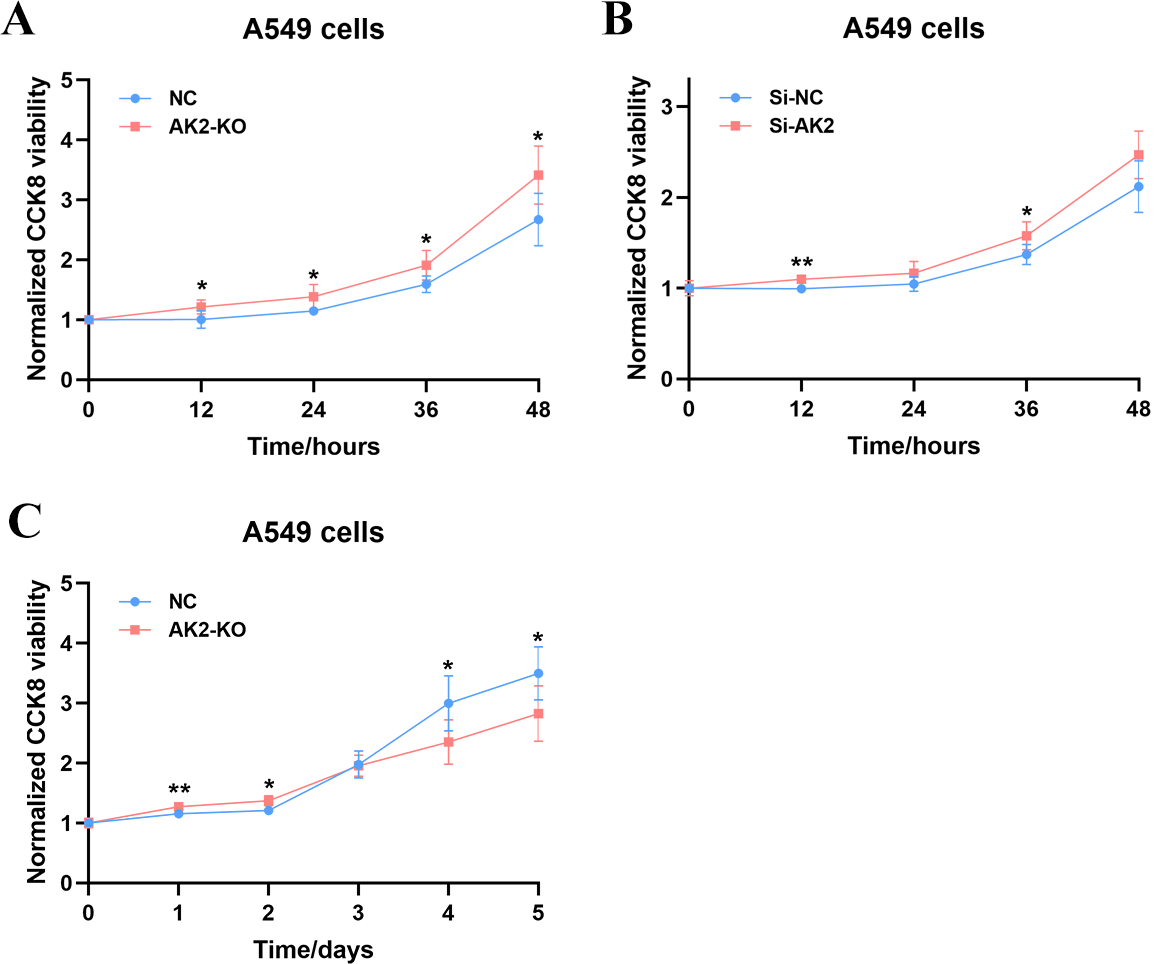


**Figure S4 Effects AK2 knockout or knockdown on lung cancer cell proliferation.** (**A-B**) CCK8 analysis of proliferation of A549 NC and A549 AK2-KO cell lines (**A**), control and AK2-knockdown A549 cell lines (**B**) within 48 h. (**C**) CCK8 analysis of proliferation of A549 NC and A549-AK2 KO cell lines within 5 days. Data are represented as mean ± S.D. **p* < 0.05, ***p* < 0.01.

**Figure S5 Effects of AK2 knockdown on lung cancer cell apoptosis.**


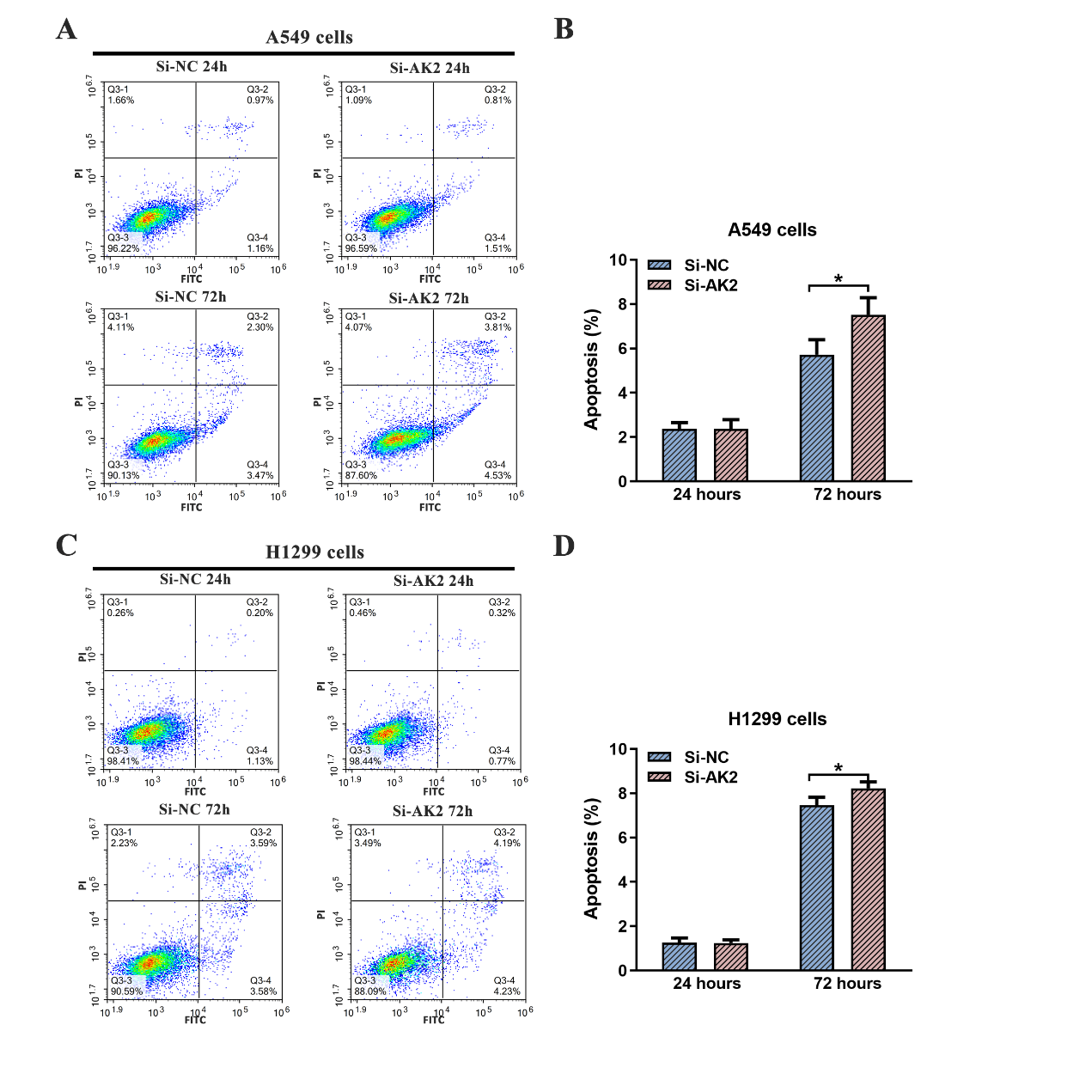


**Figure S5 Effects of AK2 knockdown on lung cancer cell apoptosis.** (**A**) Representative FACS analysis and quantitative analysis of Annexin V and propidium iodide (PI) staining of control and AK2-knockdown A549 cell lines at the time point of 24 h and 72 h. (**B**) Statistical analysis of (A). (**C**) Representative FACS analysis and quantitative analysis of Annexin V and propidium iodide (PI) staining of control and AK2-knockdown H1299 cell lines at the time point of 24 h and 72 h. (**D**) Statistical analysis of (C). Data are represented as mean ± S.D. **p* < 0.05.

**Figure S6 Knockdown of AK2 inhibits EMT process in A549 cells**.


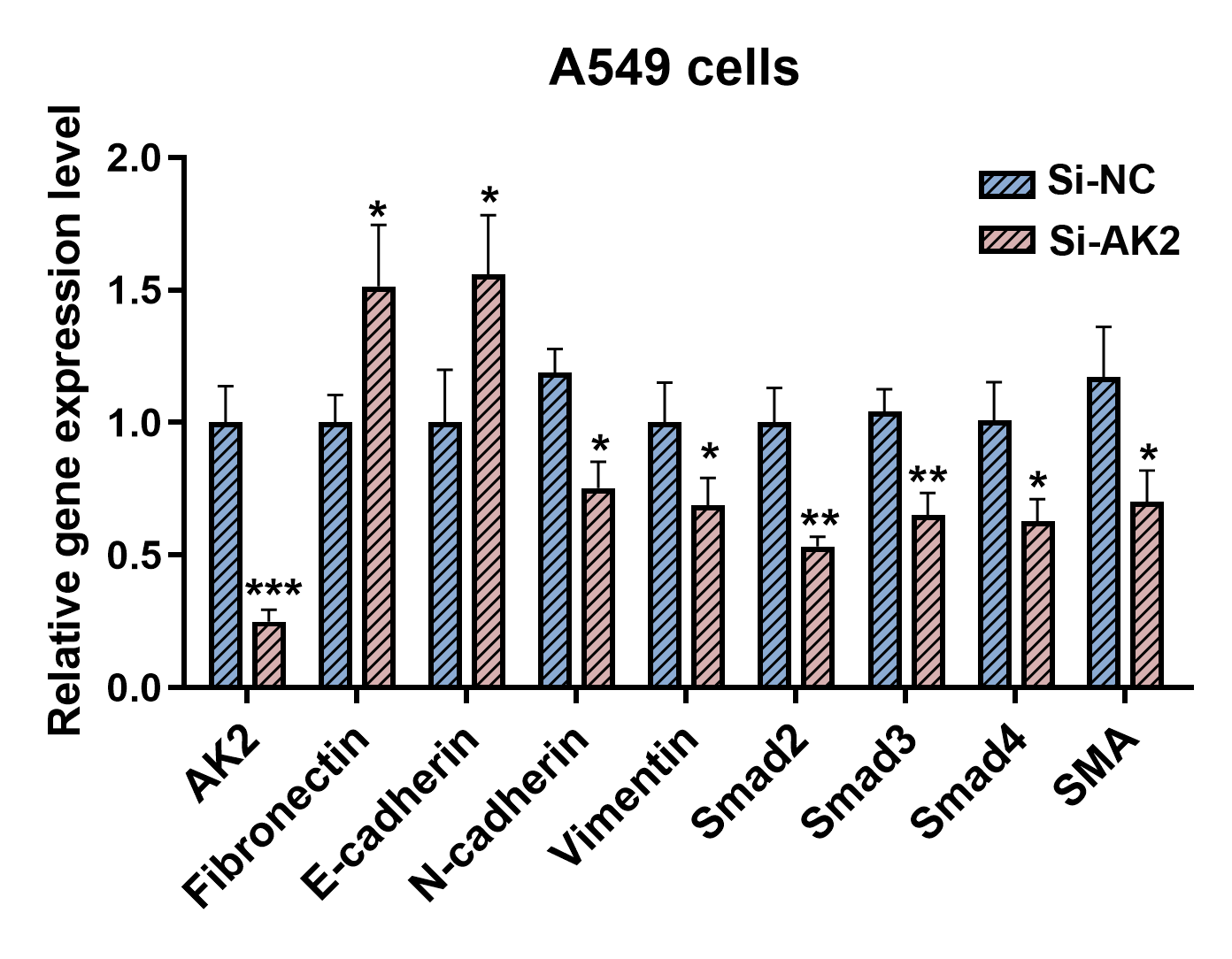


**Figure S6 Knockdown of AK2 inhibits EMT process in A549 cells**. The mRNA levels of Fibronectin, E-cadherin, N-cadherin, Vimentin, Smad2, Smad3, Smad4, SMA and AK2 were detected by qPCR analysis in A549 cells after transfection with AK2 siRNA/NC for 48 h. Data are represented as mean ± S.D. **p* < 0.05, ***p* < 0.01, ****p* < 0.005. Each bar is the mean of three independent experiments.

**Figure S7 Knockdown of AK2 inhibits EMT process in A549 cells**.


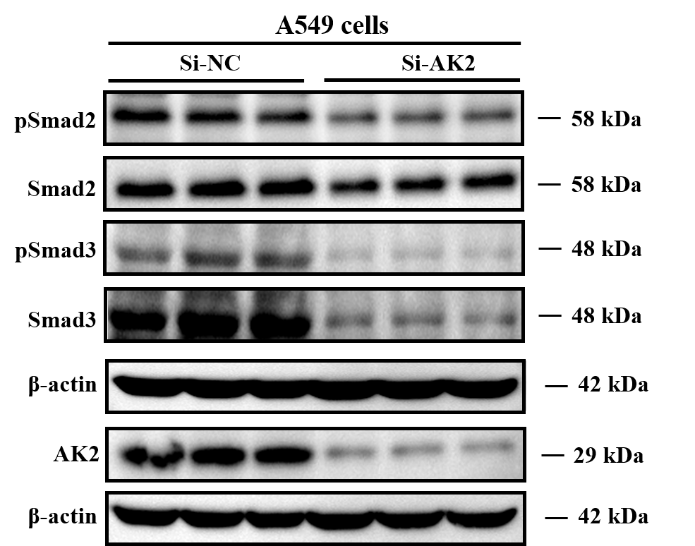


**Figure S7 Knockdown of AK2 inhibits EMT process in A549 cells**. The protein expression levels of pSmad2, Smad2, pSmad3 and Smad3 were detected by Western Blot analysis in A549 cells after transfection with AK2 siRNA/NC for 48 h.

**Figure S8 Knockdown of AK2 inhibits EMT process in H1299 cells**.


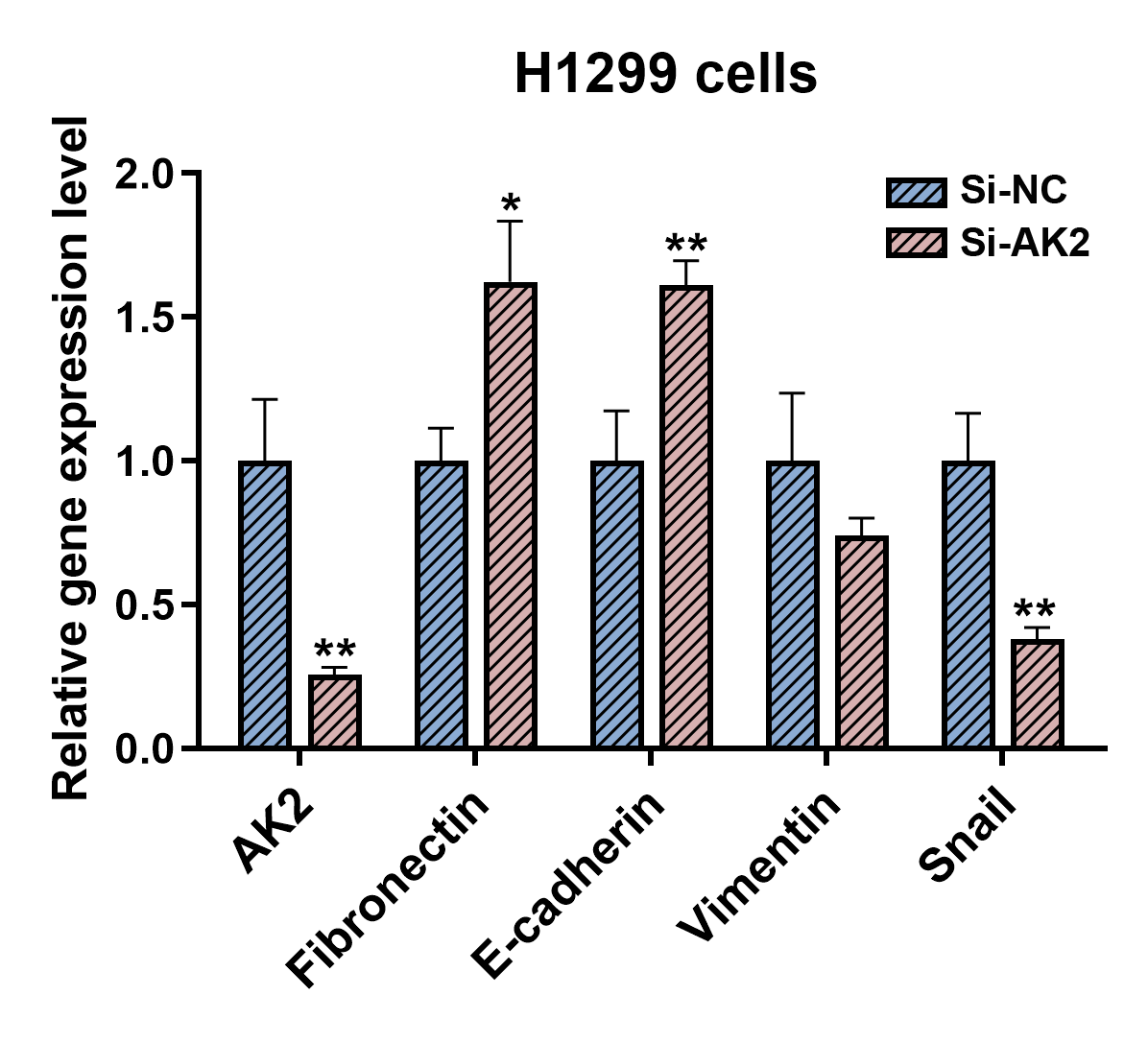


**Figure S8 Knockdown of AK2 inhibits EMT process in H1299 cells**. The mRNA levels of Fibronectin, E-cadherin, Vimentin, Snail and AK2 were detected by qPCR analysis in H1299 cells after transfection with AK2 siRNA/NC for 48 h. Data are represented as mean ± S.D. **p* < 0.05; ***p* < 0.01*.* Each bar is the mean of three independent experiments.

**Figure S9 AK2 induces Snail/Smads/TGF-β signaling to activate EMT process**.

**

**

**Figure S9 AK2 induces Snail/Smads/TGF-β signaling to activate EMT process**. H1299 cells were transfected with AK2 siRNA or NC for 24 h, respectively, and then stimulated with TGF-β for 2 h. Western blotting was performed to detect the levels of E-cadherin, Vimentin, Smad2 and AK2 respectively.

**Figure S10** **AK2 induces FADD/PKC signaling to activate EMT process**.


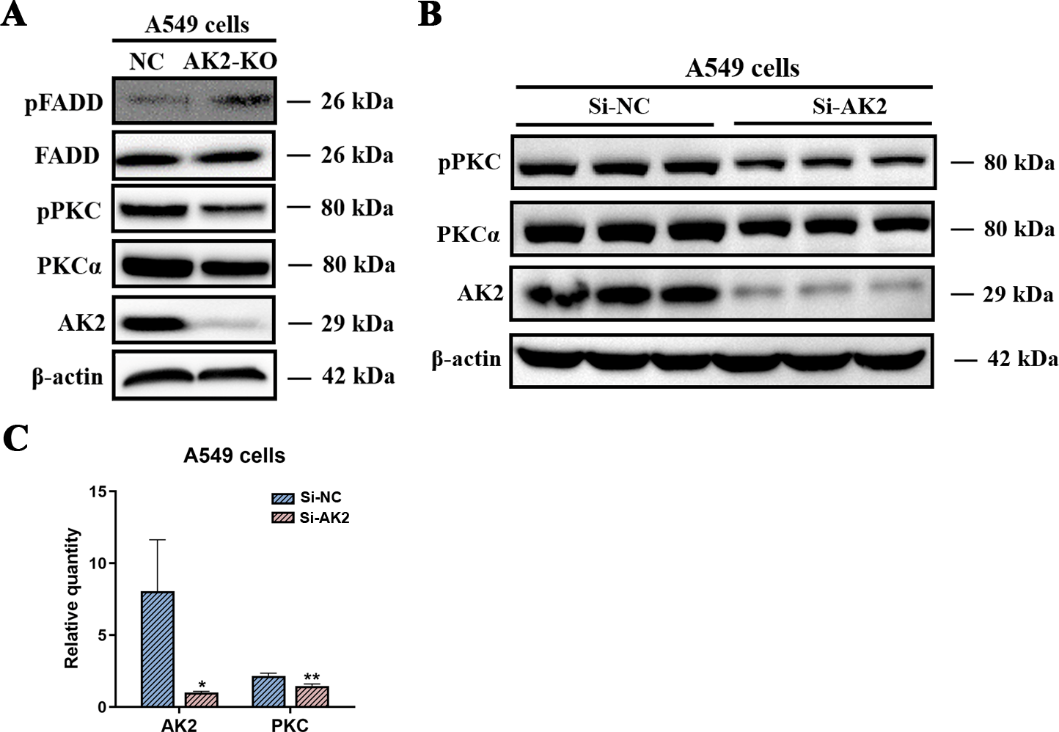


**Figure S10 AK2 induces FADD/PKC signaling to activate EMT process**. **(A)** Western blotting analysis was performed to detect the levels of pFADD, FADD, p-PKC, PKCα and AK2 respectively in control and AK2-KO A549 cells. **(B)** The protein expression levels of pPKCα, PKCα and AK2 were detected by Western Blot analysis in A549 cells after transfection with Si-NC or Si-AK2 for 48 h. **(C)** The mRNA levels of PKCα and AK2 were detected by qPCR analysis in A549 cells after transfection with Si-NC or Si-AK2 for 48 h. Data are represented as mean ± S.D. **p* < 0.05; ***p* < 0.01. Each bar is the mean of three independent experiments.
